# Prediction‐Based Algorithms for Long‐Range High‐Speed Force Spectroscopy

**DOI:** 10.1002/jmr.70043

**Published:** 2026-08-03

**Authors:** Lorenzo Villanueva, Yogesh Saravanan, Mar Eroles, Antoine Couderc, Jorge Rodriguez‐Ramos, Qingze Zou, Felix Rico

**Affiliations:** ^1^ Aix‐Marseille Univ, INSERM, DyNaMo, Turing Centre for Living Systems Marseille France; ^2^ Department of Mechanical and Aerospace Engineering Rutgers University Piscataway New Jersey USA

**Keywords:** force spectroscopy, high‐speed atomic force microscopy, long‐range interactions, prediction‐based control, real‐time algorithms

## Abstract

We present a high‐speed force microscopy platform mounted on a confocal microscope with a *z* sample stage enabling long‐range, high‐speed force spectroscopy measurements on biological samples. The control software is built on a field‐programmable gate array (FPGA)‐based data acquisition and processing system, complemented with a custom graphical user insterface (GUI). We introduce smart algorithms based on probe‐engagement prediction algorithms that leverage previously measured probe‐sample contact to accelerate the probe engagement to mm/s and decelerate it in proximity of contact to user‐defined μm/s velocities. This significantly reduces long‐range force curve acquisition time and data density. Using this system, we provide proof‐of‐concept mechanical maps of cell clusters to extract topography and viscoelastic parameters. To further explore the versatility of our system, we probed the forces required to extract membrane tethers from monocytic cells using ultrashort cantilevers functionalised with adhesion molecules. Our system allowed us to cover retract velocities from ~1 μm/s up to ~6000 μm/s, and to explore the dynamics of tether formation at physiologically relevant velocities. Our results show that coupling extended *z* displacement with the prediction‐based engagement algorithms enables rapid, quantitative mechanical mapping of heterogeneous biological samples with large topography variation and supports measurements requiring long distances at high velocities.

## 1 Introduction

AFM has become an essential tool for characterising the structural and mechanical properties of biological systems, from single molecules to cells and tissues [1, 2]. Biological models are becoming more complex in an effort to better mimic living systems, and complexity often involves large samples, in the range of tens to hundreds of micrometres thick. For example, live tissue sections from biopsies are commonly used to explore biological function and spheroids and organoids are now routinely established as a more realistic alternative to the conventional cell monolayer cultures. Mechanical characterisation of these types of samples require AFM piezoelements with long travel *z* scanners. The study of cell adhesion provides a further layer of complexity. Processes such as the leukocyte adhesion cascade involve establishment of multiple adhesion sites and the formation of membrane nanotubes or tethers, which stabilise adhesion under physiological shear flows [3]. Whole cell adhesion measurements also known as single cell force spectroscopy on different cell types with AFM have revealed tens‐of‐micrometres‐long separation distances due to the formation of membrane tethers [4, 5, 6]. These adhesion measurements require sufficient *z* piezo range so the cell can completely detach from the cantilever or another cell. AFM systems featuring long *z* scanners reaching travels of ~100 μm have been available since decades and have led to the commercialisation of long *z* scanner modules (e.g., CellDhesion) [4, 5, 7]. Some of these works specifically implemented long *z* scanners on AFM systems to probe cell adhesion [4, 5].

However, long range piezoelements are limited in actuation velocity, which leads to force–distance curves of several seconds long and, thus, time consuming measurements, incompatible with living systems. In addition, long range scanners may not reach physiologically relevant fast speeds in the mm/s range of, for example, blood flow. Moreover, the long response time of long range scanners leads to overshoot at high velocities, and thereby limits the control of the applied force. Such a limitation, in turn, hinders high‐speed force measurements on fragile biological samples. To overcome these limitations, various solutions have emerged.

Various custom and commercial AFM systems now feature a vertical range of ∼100 μm and include additional *z*‐scanners of shorter range. This allows large‐range probe displacements at moderate velocities (up to tens of μm/s), and force measurements over a limited displacement range at relatively high rates, such as oscillatory measurements upon contact [8]. This provides flexibility and widens the range of some force measurements. However, if the sample is highly corrugated, the limited response time of long range *z*‐piezo still implies low‐speed force curves, leading to force maps lasting several tens of minutes.

HS‐AFM enabled unprecedented visualisation of biomolecules at subsecond temporal resolution [9], thanks to faster electronics, rigid and miniaturised mechanical designs, and ultra small cantilevers [10, 11]. The HS‐AFM capacity of using ultrasmall cantilevers with MHz resonance frequency in liquid allowed also the implementation of high‐speed force spectroscopy (at velocities up to several mm/s, HS‐FS) and dynamic mapping of single molecules and cells at sub‐microsecond temporal resolution [12, 13, 14, 15, 16]. Many efforts have been devoted to increase the scanning speed of the nano‐positioners to achieve visual dynamics of biomolecular processes in the nanoscale by developing *z*‐scanners with resonance frequencies of several hundreds of kHz and up to the MHz range [11, 17, 18, 19]. However, a persistent limitation in current HS‐AFM platforms is their restricted vertical travel range (∼1 μm), which impedes the study of thick and highly corrugated biological samples and of cell adhesion, restricting mechanical tests at high pulling velocities to biomolecular systems [20, 21, 22, 23]. Therefore, applying HS‐AFM technology to living mammalian cells remains challenging. Unlike proteins or membrane patches, mammalian systems, such as monocytes and cell clusters, are substantially larger (10–100 μm in height) and exhibit pronounced topographic heterogeneity across regions [1]. These factors collectively demand approaches capable of fast mechanical measurements that account for such complexities in size. HS‐AFM is the expected candidate to probe large samples within reasonable acquisition times through the implementation of fast force measurements. However, new approaches are necessary to increase the actuation velocity over long travel range measurements using HS‐AFM.

Different approaches have been implemented in piezo control such as inversion based feedforward control [24] and iterative‐learning‐based control techniques [25, 26] to account for the piezo's vibrational dynamics, creep, hysteresis and response variations and model uncertainty, showing significant increase in scan frequency and range along the x–y plane. These approaches explore the a priori known and repetitive trajectories in lateral scanning and have been extended to the vertical z‐axis to track sample topography during high‐speed imaging. Recent developments have been proposed to accelerate the acquisition of scanning probe microscopy by skipping certain scan lines in x–y for then reconstructing the surface topography using deep learning approaches [27, 28]. Even though, approaches for increasing the range and speed of AFM mainly focus on improvements to AFM imaging, and only a small amount of work has been done for simultaneous hysteresis–dynamics compensation for long range force measurements.

Force mapping techniques that approximate triangular waveforms of the *z* movement to single or superpositions of sinusoidal signals effectively accelerate acquisition of mechanical maps, by reducing the excitation of resonant modes of *z* scanners [23, 29, 30]. Iterative shape compensation techniques accounting for *z* scanner's mechanical instabilities were developed for fast microrheology measurements over a large, quasi‐continuous frequency bandwidth on soft samples, importantly extending the dynamic range of *z*‐piezoelements [8, 31, 32, 33, 34]. Photothermal off‐resonance tapping also effectively accelerates the force curve acquisition time [35, 36]. Nonetheless, these techniques have been implemented at small *z* ranges (∼2 μm), only applicable to biomolecular systems or thin cell regions.

For thick samples, such as cells, recent approaches involve the continuous monitoring of the feedback signal (current in SICM or deflection in AFM) to adapt the velocity of the *z*‐scanner driving signal to decelerate near the surface, effectively allowing faster scanning velocities (up to 250 μm/s) and reducing overshoot effects [37]. The Schäffer group implemented a HS‐AFM system allowing ultrashort cantilevers and featuring a 10 μm range *z*‐scanner with 17 kHz resonance frequency driven by a low‐noise voltage amplifier which was applied to map living cells [38]. Given the fragility of the glass pipette probes used in scanning ion conductance microscopy (SICM) and its application to relatively thick samples, such as living cells, recent efforts towards acceleration of mapping capabilities have led to satisfactory results. The Fantner group developed a fast 22‐μm‐long‐range *z* piezoelectric scanner with a resonance frequency of 13 kHz in an SICM system that was successfully applied to obtain time‐resolved force maps of living cells during bacterial infection [39].

Still, performance trade‐offs remain: broad frequency coverage, long range and precise force control have been limitedly achieved simultaneously at the higher scanning speeds (hundreds of Hz or a few μm/s). For soft, heterogeneous samples such as live cells, balancing accuracy and throughput thus remains a significant challenge. This constraint also applies especially to cell adhesion experiments to study processes such as the leucocyte adhesion cascade [3, 40, 41], where exploring adhesion at physiologically relevant retraction velocities in the millimetre‐per‐second range demands a long vertical travel to reach and maintain those speeds while still capturing high‐temporal resolution force curves.

The aim of this work is to develop a force spectroscopy platform based on smart algorithms to accelerate the actuation velocity of long‐range force measurements. We present optimised algorithms designed for time‐ and data‐efficient broadband nanomechanical mapping integrated into an extended *z*‐range HS‐AFM system. As proof of concept experiments, we applied these developments for the rapid characterisation of the mechanical heterogeneity of thick cell clusters and to probe leucocyte adhesion at physiologically relevant fast pulling velocities.

## 2 Materials and Methods

### 2.1 HS‐AFM System

Measurements were performed using a modified probe scanning HS‐AFM system (PS‐NEX, RIBM, Japan), mounted on the stage of an inverted optical microscope (Ti2 Nikon, Japan) and coupled to a spinning disk confocal microscope (CSU‐X1, Yokogawa, Japan and camera Prime 95B, Photometrix, Tucson, AZ). The HS‐AFM head contains the optical lever detection system, the laser diode and focusing optics, a two segment photodiode and the cantilever holder. The XY movement was carried out through the stage piezoelements (45 × 45 μm) and was directly controlled from the incorporated data acquisition board through the original piezo amplifier box (M‐2629, MESS‐TEK LTD). Though the original use of this microscope was tapping mode imaging, the system was controlled for force measurements using external FPGA acquisition boards (7976R and 7856R, National Instruments, Austin, TX). The control system is composed of a NI PXI 1081 chassis connected to the host PC via PCIe card and the two FPGAs interconnected via hardware with dedicated communication buses and synchronised by an internal 40 MHz clock, or 0.025 μs per clock cycle (1 tick).

We designed a long range 3D printed (Polylactic Acid, PLL, Formlabs, Sommerville, MA) hinged *z*‐stage adapted to the XY piezo stage frame. The *z*‐stage houses a piezo actuator (P603.1S2) with a nominal displacement of 100 μm, strain gauge position sensor and controlled by a dedicated piezo amplifier (E709.604, Phisik Instrumente, Germany). The *z*‐stage is built through a hinge‐like geometry. Therefore, the effective output movement was reduced. Calibration of the *z*‐stage response was carried out through two different methods, by determining the change in the focusing of the sample upon a step of the *z*‐scanner and through the slope of a force curve on a hard substrate. The two methods led to similar results and a maximum travel range of 60 μm.

Given the open loop operation of the XY scanner, we enforced a unidirectional trajectory for the XY mapping, thus scanning the slow *X*‐axis from right to left for each line. The calibration of the XY displacement was carried out optically through the known pixel size of the coupled camera.

Two component removable glue (F20, Plastiform, France) was used to mount the cantilever into the custom‐made cantilever holder. A liquid sample holder was designed and 3D printed with a beveled square interior angle to hold liquid up to 0.8 mL, while maintaining a liquid meniscus and safe distance from the cantilever head.

### 2.2 Control Algorithms and Graphical User Interface (GUI)

A GUI was designed and built to pilot, read and save data from the HS‐AFM, to operate the long *z* range piezo, and the FPGA software to implement force feedback, PID and the algorithms described in the results section. HS‐AFM control software was developed in LabVIEW and included the GUI and FPGA bitfile.

### 2.3 AFM Measurement and Analysis

Force measurements were carried out using different cantilevers: ultra small AC10 (resonance frequency $f_{R}$: 491 kHz, spring constant k: 0.135 N/m, invOLS: 18.65 nm/V), AC40 ($f_{R}$: 24 kHz, k: 0.069 N/m, invOLS: 22.5 nm/V) (Olympus, Japan) and precalibrated PFQNM‐LC ($f_{R}$: 24 kHz, k: 0.113 N/m, invOLS: 34.50 nm/V) (Bruker, Santa Barbara, CA). The spring constant was calibrated in air using the Sader method (precalibrated for PFQNM) and the optical lever sensitivity was determined from the thermal spectrum in liquid obtained with the probe at least 100 μm away from the surface [41, 42]. All measurements were conducted in liquid.

Force curve files were saved in .tdms format containing metadata on the AFM system and force curve measurement parameters. Elastic or viscoelastic characterisation from force curves, including topography, Young's modulus and fluidity index, were determined using PyFMlab [43]. Curves with failing and non‐converging fits were not considered (blank pixels in maps). The tether data analysis and force maps reconstruction were carried out using custom software written in Python 3.9 and libraries NumPy, SciPy, Matplotlib, lmfit and afmformats [44, 45, 46, 47].

### 2.4 Cell Culture

Human monocytic cells (THP‐1) were cultured in RPMI‐1640 (Sigma‐Aldrich, Merck) medium supplemented with 10% foetal bovine serum and penicillin—streptomycin (50 U/mL—50 μg/mL) at 37°C with 5% CO_2_ and humidity. Cultures were maintained by adding fresh medium every 2 days. Complete medium renewal was performed once a week, cells were then counted and subcultured at 1–2 × 10^5^ viable cells/mL. For AFM experiments, cells were collected and centrifuged. Supernatant was discarded and cells were re‐suspended in fresh serum‐free medium. After a second centrifugation to get rid of traces of FBS, cells were again suspended in serum‐free medium and seeded on glass slides for at least 30 min at 37°C to let them stick. During measurements, we used serum‐free medium supplemented with 50 mM HEPES primarily to wash away detached, floating cells while retaining immobilised cells [48, 49].

Circulating tumour cells (CTC‐44) were derived from a colorectal cancer patient and established by the team of Julie Pannequin at the Institut de Génomique Fonctionnelle (IGF, Montpellier) [50]. CTC‐44 were cultured in DMEM (Thermo Fisher) supplemented with decomplemented Fetal Bovine Serum (Thermo Fisher, 10% final), l‐glutamine (2 mM final) and penicillin—streptomycin (100 U/mL—100 μg/mL) at 37°C with 5% CO_2_ and humidity. At 80%–90% confluency, cells were passed by eliminating supernatant, the monolayer was gently rinsed with PBS, and the same volume of trypsin was added. After 3 min of incubation at 37°C, complete medium was added (twice the volume of trypsin) to inactivate the trypsin. Cells were then counted and seeded in a new flask at a 150,000–200,000 cells/mL density. Cell clusters grown in 3D were then transferred to poly‐l‐lysine coated glass slides and incubated for at least 1 h at 37°C before experiments.

### 2.5 Cantilever Functionalisation

For tether measurements, AC10 cantilevers were coated with human vascular cell adhesion molecule 1 VCAM‐1 Fc chimaera (huVCAM‐1, R&D systems 862‐VC), using a protocol described before [5]. Briefly, cantilevers were cleaned by rinsing in Milli‐Q water, followed by UV irradiation (5 min) and immersion in acetone (10 mL). After drying under a nitrogen stream, cantilevers were activated by UV/ozone treatment (15 min). Surface silanisation was performed by incubating the cantilevers in 5% (v/v) APDMES in absolute ethanol for 10 min at room temperature, rinsing with ethanol, drying under nitrogen and curing at 80°C for 30 min. The silenised cantilevers were subsequently incubated in 0.1% glutaraldehyde in 1× PBS for 30 min at room temperature, then washed five times in fresh PBS and rinsed in Milli‐Q water to avoid salt crystallisation. Cantilevers were dried by transferring across dry wells. For protein immobilisation, dried cantilevers were incubated in 25 μg/mL VCAM‐1 solution for at least 1 h at room temperature or overnight at 4°C in a humid chamber. After coating, cantilevers were transferred to tris‐buffered saline (TS) and stored at 4°C for up to 7 days. Prior to use, cantilevers were incubated in TS containing 0.01%–0.1% BSA for 10–30 min to block residual aldehyde groups and reduce nonspecific adhesion. While immobilising the cantilever on the holder, moist Kimtek tissue with milliQ water was placed close around to prevent drying.

## 3 Results

We developed a prediction‐based force–distance curve algorithm, named ‘moustache’,^1^ designed for time and data efficiency of high‐speed force spectroscopy applications requiring large‐displacement ranges. The algorithm allows rapid, velocity‐controlled probe approach and retraction up to mm/s. The algorithms controlled a home‐built high‐speed AFM with a large scan range of 45 μm × 45 μm in xy‐plane and 60 μm in the vertical z‐axis direction. The system allows the use of ultrashort HS‐AFM cantilevers with μs‐response time and high‐speed approach and retract speeds up to ∼6 mm/s.

**FIGURE 1:**
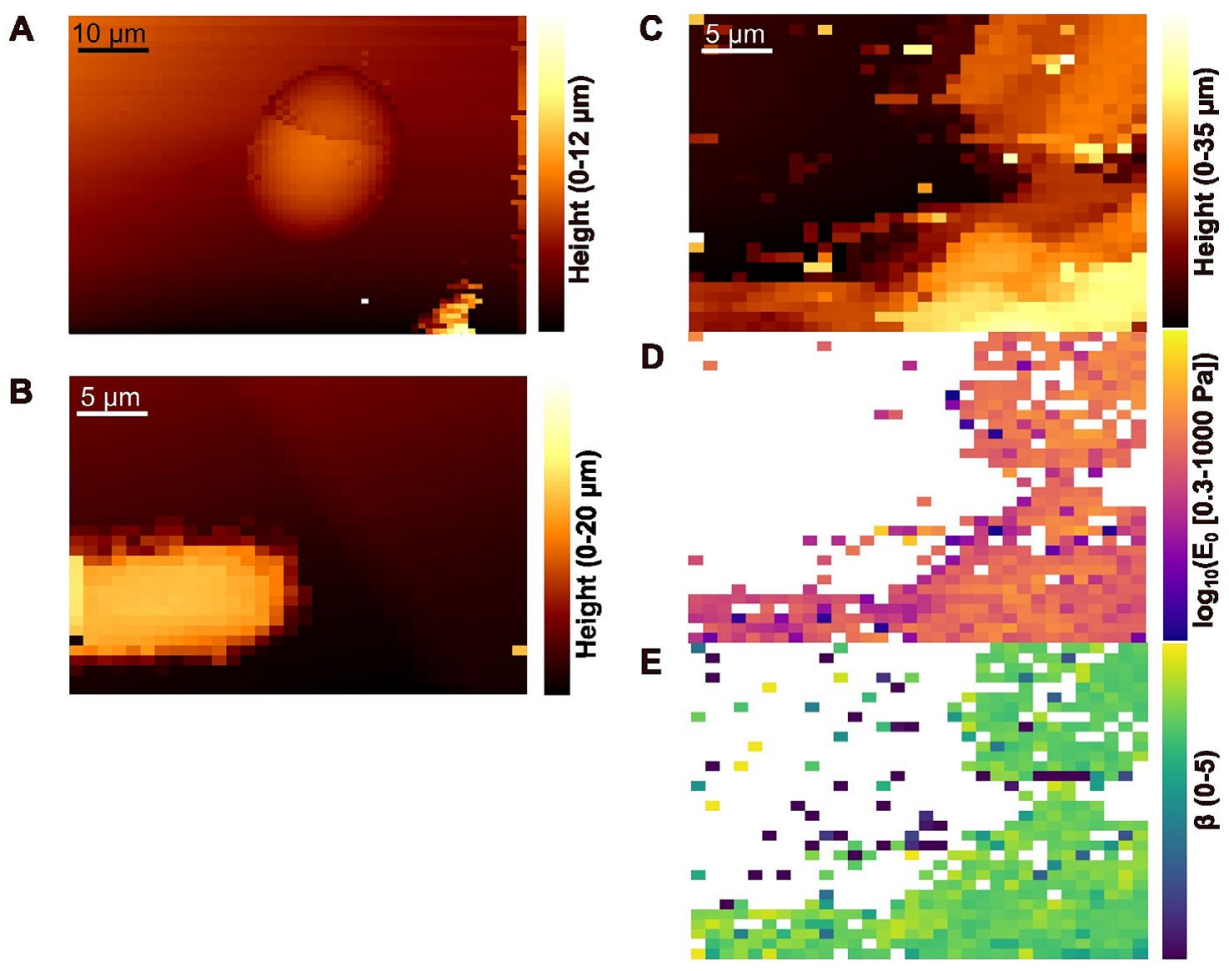
(A) 64 × 64 topography map of 10 μm polystyrene beads. Beads were melted on a glass slide and measured in liquid with an AC40 cantilever. (B) 32 × 32 topography maps of THP‐1 cells acquired in liquid with an AC40 cantilever. (C–E) 32 × 32 grid over a 24 × 17 μm^2^ area using a *z*‐piezo range of ∼60 μm map of CTC‐44 force‐indentation acquired with the ‘moustache’ curve algorithm, analysed in PyFMLab: (C) topography, (D) elastic scaling factor, $E_{0}$ and (E) fluidity index.

To test the home‐built long range *z*‐piezo stage of the high‐speed AFM system, we first mapped various samples using conventional force curves (Figure 1A–E). The first map was obtained on a polystyrene bead partially melted on a glass surface. The geometry and dimensions correspond to the expected flattened shape of the 10 μm bead. Next, we mapped the topography of an THP‐1 isolated monocytic cell slightly immobilised on the surface. Again, the obtained topography was in agreement with the reported ∼15‐μm diameter of this cell line [27]. Finally, we mapped clusters of circulating tumour cells (cell line CTC 44) with a topography range of ∼40 μm. Each force curve was fitted with a viscoelastic contact model to extract the scaling elastic modulus (or rigidity) and the fluidity parameter.

### 3.1 Prediction‐Based *Moustache* Rapid Force Curve Algorithm

**FIGURE 2:**
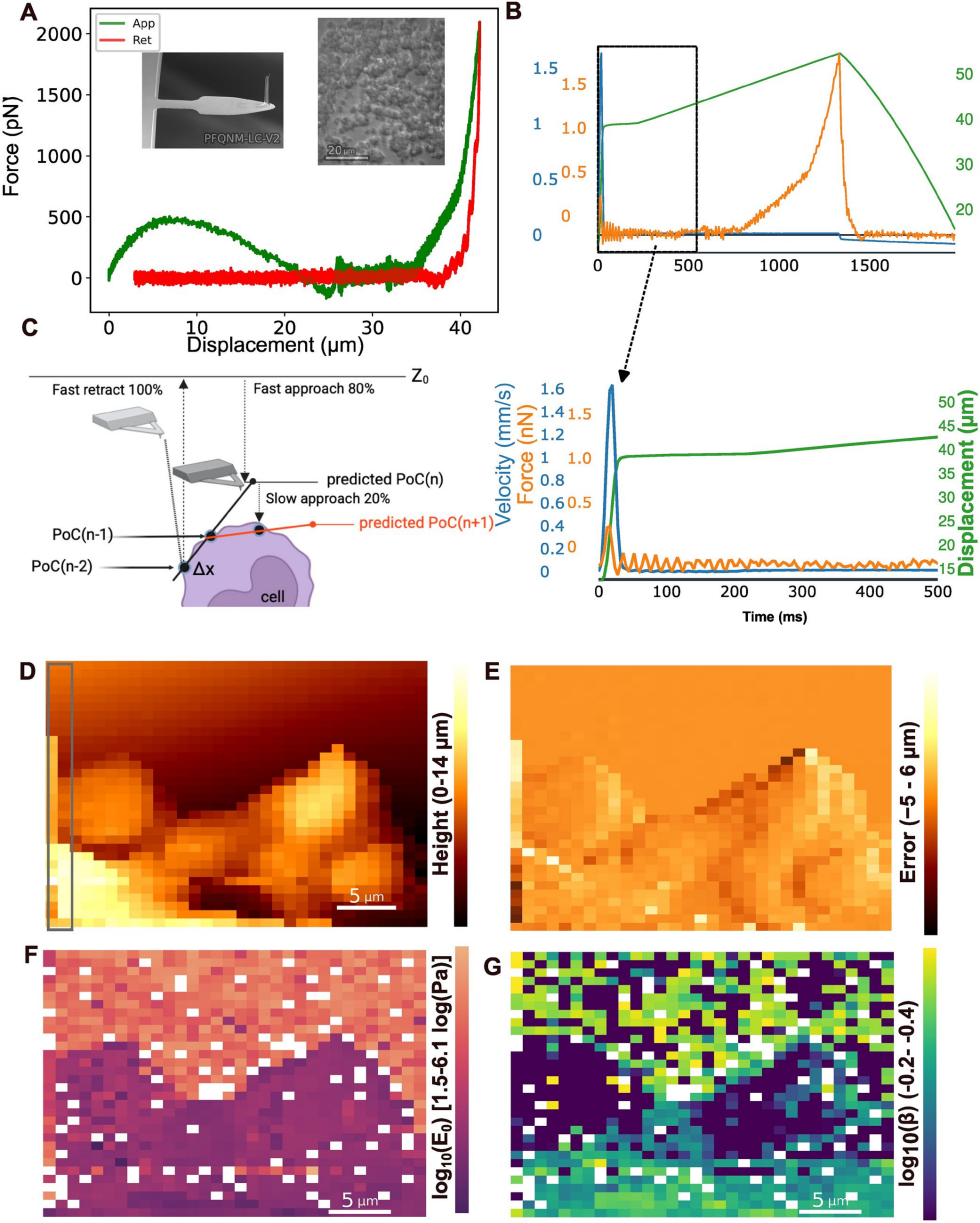
(A) Long‐range force–distance curve acquired on a CTC‐44 cell cluster using the moustache algorithm. The green line shows the approach segment and the red line the retract segment. The insets show an electron microscopy image of the used PFQNM cantilever (left) and a bright field image of the cell sample (right). The total *z* travel was 60 μm. (B) Top: Same force curve as in (A) plotted versus time, showing *z*‐piezo position (green), cantilever deflection/force (orange) and *z*‐piezo velocity (blue). Bottom: Zoom of the fast‐approach segment (boxed) showing a 1.5 mm/s peak approach velocity, travelling ∼35 μm in ∼50 ms. (C) Diagram of the predictive topography algorithm using linear‐interpolation to estimate the next contact point along the *x*‐axis during force‐mapping. The sequence begins by acquiring an initial force curve at a low velocity (∼50 μm/s) to robustly locate the first point of contact PoC(*n* − 2). For the subsequent force curve, the probe rapidly approaches ∼80% travel distance of the previous contact point, then decelerates to the target velocity to reach the surface and define the new PoC. Each detected PoC is stored and all subsequent PoC is extrapolated using the last two PoCs. After each approach force curve, the *z*‐stage returns to a starting position $z_{0}$ (here $z_{0}$ = 0; $z_{0}$ may vary with sample/scan settings). (D) Topography map of the CTC‐44 cluster acquired using the predictive algorithm. (E) Error topography map, illustrating the predicted‐height bias: pixels located between two height peaks tend to yield underestimated contact heights, and the converse occurs at local minima. (F) Elastic scaling factor ($E_{0}$) map. (G) Fluidity index map.

The moustache algorithm explores the a priori knowledge of the approximated sample surface position relative to the tip, estimated based on the measurements at preceding sample locations. This allows the tip to initially approach at high velocity and then transit to a well‐controlled, slow velocity for the final tip‐sample engagement (Figure 2A–C). In the first step, an initial force curve at a relatively slow velocity, typically 50–100 μm/s, is acquired to detect the initial sample surface point of contact (PoC). Using this reference on the consecutive force curve, the cantilever rapidly approaches the surface at high velocity (up to the maximum velocity allowed by the *z*‐scanner before causing exceeding oscillations) for the most of the total displacement range at the preceding PoC (e.g., 80% of the total expected range to the contact point). The approach then is switched to a slower velocity to reach the desired force setpoint with a well‐controlled engagement velocity, minimising the risk of probe and sample damage. Finally, the cantilever is retracted at the desired velocity, up to ∼6 mm/s. The moustache force curve reduced the acquisition time by a factor of ∼10, also reducing the data density and, thus, the volume of the corresponding force curve files. To prevent possible damage during this fast approach, the force was monitored in real time and, if the setpoint was reached within the chosen (e.g., 80%) travel distance, the tip was immediately retracted. To minimise the excitation of the resonant frequency of the *z*‐piezo element (∼1 kHz), the beginning and end regions of the fast approach were rounded using an IIR second order low‐pass Butterworth filter with a cutoff frequency of ~175 Hz. To maximise the high‐speed capabilities, we operated the *z*‐piezo element in open‐loop mode during the high velocity movements.

The developed algorithm operates using an FPGA loop at 500 kHz, corresponding to a 2 μs cycle time, with a fixed velocity of 6 μV/s, developing a maximum velocity of ∼1.5 mm/s. The first fast approach segment, ranging from 0 to 1, is stored in a look‐up table (LUT), accessed by the FPGA loop and scaled by the 80% of the required travel range (safety movement). The second *slow* approach segment is calculated at each timestep based on the previous position of the *z*‐scanner and the set velocity, minimising memory usage. At each FPGA cycle step, a *z* value was fed into the while loop and added to the previous position. Certain transitions such as changing the fast approach to normal approach velocities were processed in the GUI's backend. A lag of 200 ms occurs when switching from the fast approach to the controlled normal approach. This delay could be minimised by optimising the FPGA state logic for faster transitions. Nonetheless, this transient time allows for stabilisation of the cantilever ringing after the fast approach segment.

A representative example of a moustache force curve acquired on a cell cluster at a fast approach velocity at 1.5 mm/s is shown in Figure 2A. The first ∼25 μm reveal the high viscous drag force (∼750 pN) exerted on the cantilever due to friction (viscous drag coefficient ∼0.5 pN/nm s, AC40) that decreases once we reach 35 μm and the tip approaches the surface at 10 μm/s until a setpoint of 1.5 nN. Finally, the sample retracts at ∼100 μm/s velocity. This whole force curve cycle took ∼2 s, shortening the time and data density on redundant regions for mechanical analysis. Figure 2B shows the same force curve over time. The retract segment, in green, reveals the nonlinear piezo movement observed due to open loop piezo operation.

### 3.2 Point of Contact Prediction Algorithm

The moustache algorithm efficiently reduced the acquisition time and data volume of the force curve and was effective on a fixed x–y position, as the point of contact was not expected to change importantly from one force curve to the next. To apply the moustache algorithm while scanning in x–y on corrugated surfaces, knowledge of the PoC at the next pixel is required. Consequently, we developed a second algorithm to predict the surface topography (Figure 2C). In the prediction algorithm, at each map line along the slow scanning axis (x), the point of contact of the *n* pixel (PoC(*n*)) was updated dynamically by linear extrapolation from the last two points of contact

$$\mathrm{PoC}\left(n\right)=\mathrm{PoC}\left(n-1\right)+\left(\mathrm{PoC}\left(n-2\right)-\mathrm{PoC}\left(n-1\right)\right).$$

The first force curve of each line was carried out at a moderate velocity to detect the point of contact, and the second force curve used the first PoC using the moustache algorithm. Then, the force curve at subsequent pixels followed the moustache algorithm with the safety movement of 80% of the travel upon the predicted PoC(*n*) and completely retracted after contact. This guaranteed efficient scanning while maintaining safe lateral movement and precise interaction with the sample surface.

### 3.3 Mapping of Cell Clusters

As a proof of concept of the prediction algorithm, we mapped clusters of circulating tumour cells with an expected thickness ranging up to ∼40 μm. We covered an area of 30 × 40 μm^2^ with 32 × 32 pixels, using the full range of the *z*‐piezo (∼60 μm). We used a fast approach velocity of ∼2000 μm/s, a slow approach velocity of ∼10 μm/s and retract velocity of ∼100 μm/s. The average time of a force curve was 1.5 s, while using a constant approach and retract velocity of 10 μm/s, it would be of ∼10 s. Therefore, the data acquisition was ∼7 times faster. For a map of 32 × 32 pixels, this represents a total acquisition time of ∼25 min, instead of ∼2.8 h, nonviable for the study of living cells. In addition, the size of the stored data was of 0.6 Gb, about 10 times lower than using conventional approaches, and reasonably tractable, storable and shareable.

**FIGURE 3:**
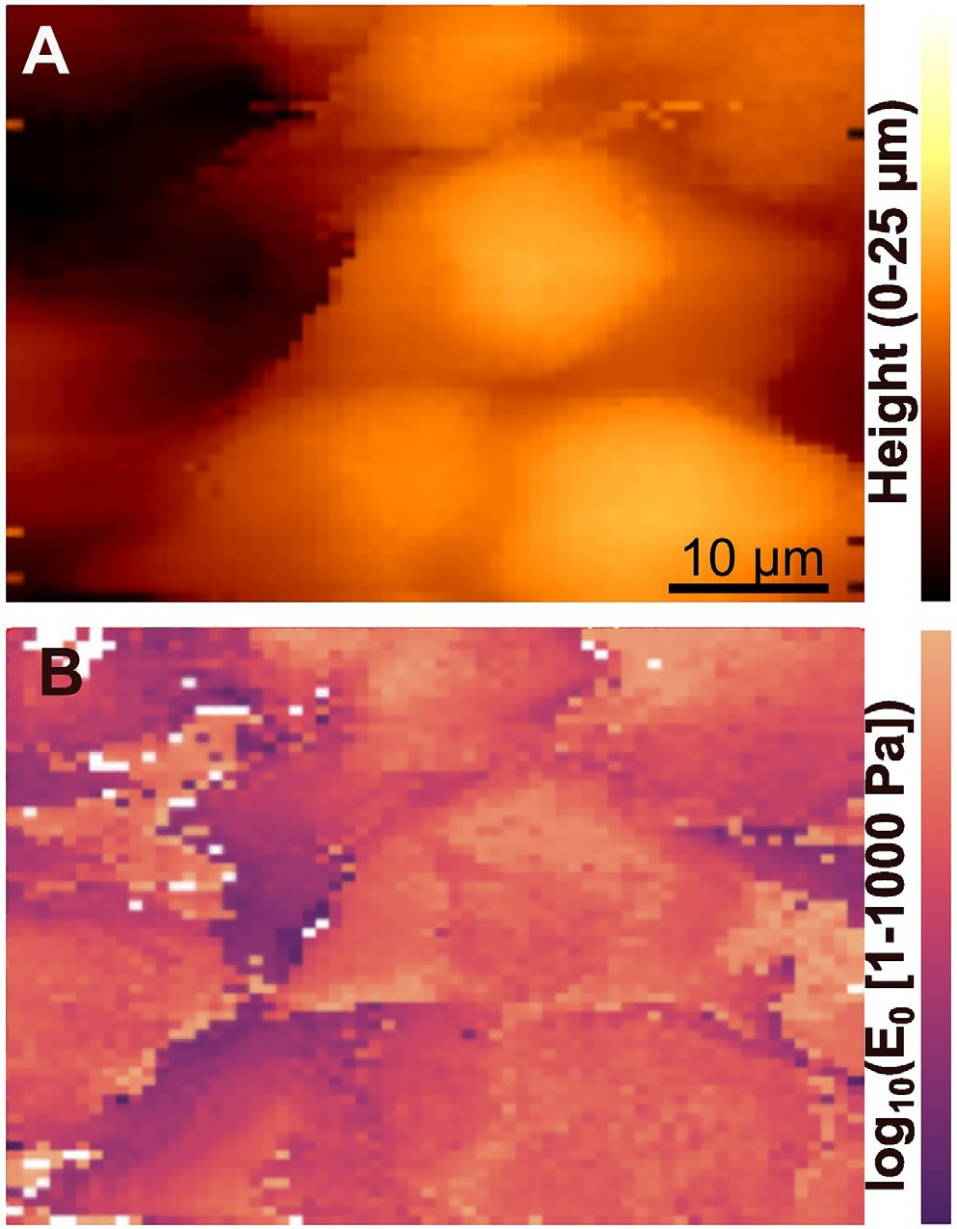
(A) Representative example of a CTC‐44 cell cluster height and (B) Young's modulus at 64 × 64 pixels.

Figure 2D shows a representative example of the obtained topography map from the fast approach curves that was reconstructed back to back along with the predicted topography. The error map showing the difference between the predicted and actual topography reveals that ~95% of the differences were below 2 μm, around 15% of the sample height (Figure 2E). The prediction algorithm faithfully renders the topography with just small deviations mainly at the beginning of each line (highlighted by the rectangular box) and near abrupt topography changes. The former corresponds to the first two lines of the predicted topography, since the algorithm requires at least two points to calculate the next point; while the latter, to the overestimation between valleys of heights. Even in the few cases of prediction discrepancies, the algorithm was capable of responding fast to avoid crashing the tip, thanks to the 80% safety movement. The slow approach revealed force curves suited for extracting the viscoelastic properties using a viscoelastic contact model [43]. The resulting stiffness and fluidity maps are shown in Figure 2F,G, respectively. In addition, we acquired maps of cell clusters at higher resolution 64 × 64 pixels, revealing more detailed features of the cell topography and Young's modulus (Figure 3). This proof of concept experiments showed that the proposed algorithms allowed us to accurately extract topography and viscoelastic properties of living biological samples within reasonable time and with accurate control of the applied forces, while exploiting the full 60‐μm range of the *z*‐scanner.

### 3.4 Tether Dynamics at Physiologically Relevant Fast Velocities

To further test the capabilities of the HS‐AFM system and the versatility of the developed algorithms, we applied it to cell adhesion, particularly, to study the formation of membrane tethers on leucocytes at fast, physiologically relevant velocities. Membrane tethers on leucocytes may extend over several tens of micrometres, requiring long range piezoelements. However, the only relevant segment from the force curve to analyse is the retract trace. Therefore, the information contained in the approach curve is mostly irrelevant and only required to contact the cell. In addition, tether formation within the blood stream occurs at mm/s velocities over several micrometres. While the use long range piezo element features a relatively fast response, it is still limited to ∼1 ms. Therefore, moving at mm/s velocities induces an overshooting of at least 10 μm, effectively covering the whole range of the *z*‐scanner. Therefore, the proposed moustache algorithm is optimal for this kind of measurements, as it allows minimising the time of the unused approach trace, while allowing for long range retraction over a wide range of velocities. To test it, we used the monocytic cell line THP‐1, which expresses the VLA‐4 integrin (α4β1). When pulled through a vascular cell adhesion molecule (VCAM‐1), the engagement of the VLA‐4/VCAM‐1 bond often induces the formation of membrane tethers, which allows rolling of monocytes under flow [5, 39]. THP‐1 cells were immobilised on a glass slide and the ultrashort cantilever was functionalised with VCAM‐1. Measurements were carried out in serum‐free cell culture medium. We then applied the moustache algorithm varying the retraction pulling velocities from ∼3 μm/s to ∼6000 μm/s, reaching velocities ~50 times faster than in previous tether experiments [51]. To confirm that tether formation was mediated by VCAM‐1, we carried out control experiments by adding soluble VCAM‐1 (at 0.1 μg/mL final concentration) to the measurement buffer. After 20 min of incubation, the binding frequency effectively decreased from 63% to 23%, revealing the specificity of VCAM‐1 mediated tether formation.

**FIGURE 4:**
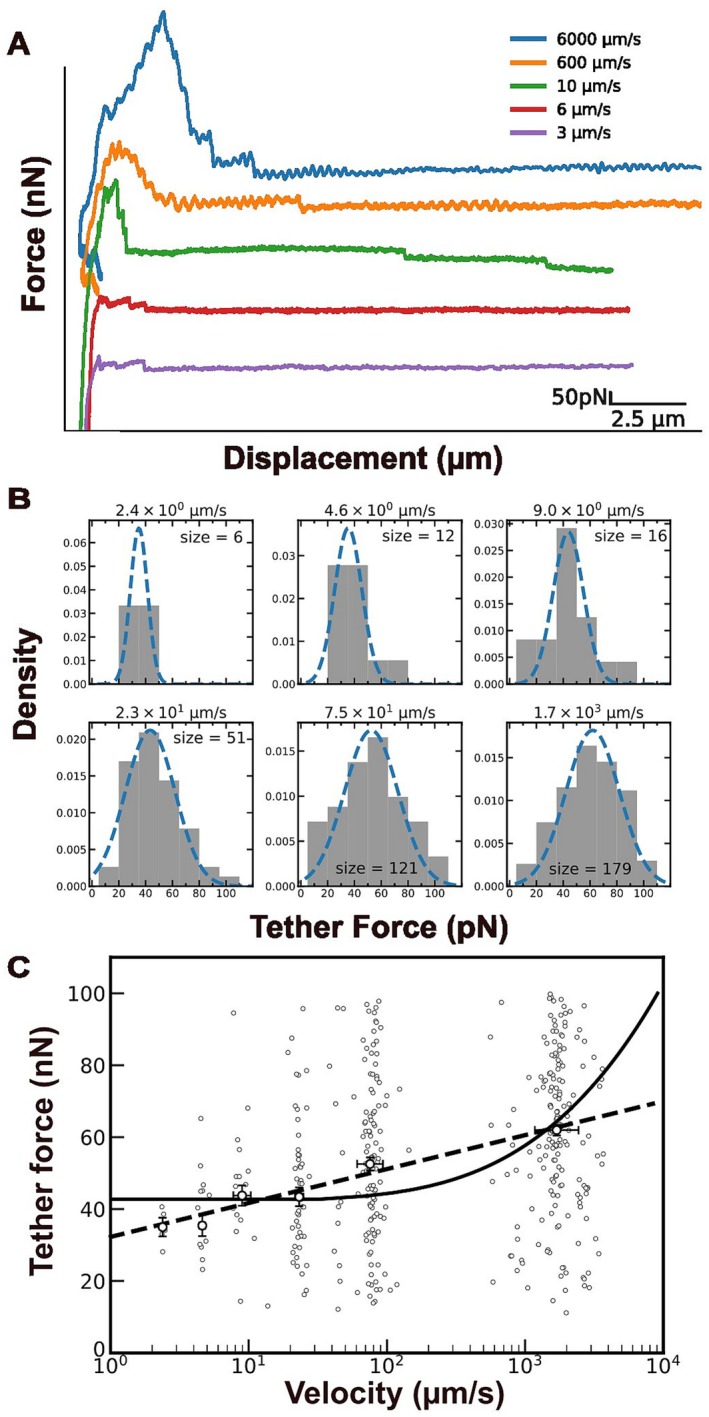
(A) Retract segment of various force–displacement curves at divergent velocities, showing tether events, evident as plateaus followed by sharp force steps. (B) Histograms of tether extraction forces at six different velocities (grey bars). A Gaussian fit (blue, dashed line) was used to determine the most probable force (vertical, blue, solid line). (C) Most probable tether extraction forces versus retraction velocity ranging 3–6000 μm/s. The solid line is the best fit of the Brochard–Wyart model and dashed line is a logarithmic fit.

Representative examples of force curves at various velocities are shown in Figure 4A. Retraction curves revealed strong adhesion forces at short pulling distances followed by one or more force plateaus, indicating the formation of 1–15‐μm‐long membrane tethers. The last step force was quantified for all successful tether forming events at the different velocities. Tether forces were pooled by velocity and histograms were then generated (Figure 4B). From each histogram, we extracted an average tether force using a Gaussian fit, which was then plotted as a function of pulling velocity (Figure 4C). As reported before, the tether force increased with pulling velocity with a slight upturn at the highest pulling rates. We used the model proposed by Brochard–Wyart to describe the experimental results, which predicts that the tether force is constant up to ∼100 μm and then increases with the cubic root of the pulling velocity ($\overset{`}{L}$) [51]. A Bell–Evans logarithmic fit was also applied as it seemed to better describe the results (see Section 4).

## 4 Discussion

We have implemented a force spectroscopy platform based on smart algorithms for HS‐AFM force measurements that require long *z*‐travel ranges. As proof of concept experiments, the platform has been applied to map highly corrugated clusters of cells and to probe the forces required to extract long membrane tethers from leucocytes over a wide dynamic range. The proposed moustache algorithm allows reducing the acquisition time and volume of data of each force curve by ∼10‐fold. Indeed, conventional AFM scanners for cell measurements feature 10–20 μm travel ranges. Operating at tens of μm/s, they require ∼1 s to travel their full range. Scaling to a larger‐travel piezo (e.g., 100 μm) implies ∼10 s per full travel over heterogeneous topographies. In the context of force mapping naively covering the full range at each pixel would be prohibitively time‐consuming, often extending acquisitions to hours for heterogeneous samples, limiting the applicability to living systems. To address this, the moustache algorithm combines knowledge of the sample topography with a fast‐to‐slow approach strategy. By estimating the local sample height, the controller executes rapid approaches when the predicted clearance is large and transitions to a slower, protective regime as the tip nears the anticipated surface. This adaptive approach reduces the dwell associated with unnecessary long‐range travel, substantially reducing by ∼10‐fold the acquisition time and data volume of individual force curves. Importantly, the approach preserves continuity with high‐velocity approach/retract segments, enabling access to higher dynamic loading/unloading regimes. Collectively, these algorithms improve throughput for heterogeneous samples while maintaining the mechanical fidelity expected from AFM‐based force measurements.

In the current configuration, the moustache algorithm includes a fast retract segment operated in open loop. This may limit the application of viscoelastic contact models that require fitting the approach and retract curves on well defined, controlled and linear approach and retract indentation. The algorithm is, however, easily adaptable to include a slow, closed‐loop approach and retract segments over a short distance, respectively preceded and followed by fast approach and retract until complete withdraw. Including feedforward mechanisms to linearise the piezo displacement in open‐loop operation would also further improve the present form of the algorithm. Finally, different types of piezo displacements may be included, such as chirp oscillation during contact or force clamp control [8, 52].

The PoC prediction algorithm extends the applicability of the moustache algorithm to force maps. This simple algorithm uses the topography values from the two previous force curves (pixels) in a map to estimate the point of contact of the current pixel, at which the fast, moustache approach algorithm is applied. Given the time and data volume reduction of the moustache algorithm, a ∼10‐fold reduction is also applied, by extension, to force maps. This is particularly important to reduce the measurement time of large living samples, such as cell clusters or living tissue sections. The algorithm presents, however, some limitations. During mechanical mapping, applying a small displacement between pixels along the scanning axes is important to accurately predict the next PoC, especially for highly corrugated, non‐homogeneous surfaces, as lateral displacement may lead to crashing the probe. In the example shown in Figure 2, with a pixel size of ~1.2 μm, the difference between the predicted and the actual topography was less than 2 μm for a total sample thickness of ~14 μm (15% difference). This supports our conservative choice of 80% safety movement of the fast segment upon PoC(*n*) of our algorithm. Nevertheless, if the tip is completely retracted at the end of each force curve, there is no possibility of crashing the AFM tip during lateral movement. In addition, since the force is continuously monitored and most topography changes may occur on the soft biological system and not on the hard substrate, even for non‐accurate determination of the next PoC, the algorithm prevents crashing of the tip over the surface and overshoot effects due to finite response time of the *z*‐piezo (~1 ms) are importantly minimised. The prediction algorithm may be improved by using all the previous points in the scanning axis and non‐linear extrapolation or considering the XY topography coupled to a 2D prediction algorithm. While more precise, these improvements may overload the computational capacity of the FPGA card without significantly improving the usability of the algorithm.

The force maps obtained using the moustache and PoC algorithms (Figures 2D–G and 3A,B) provided information about the mechanical properties of clusters of circulating tumour cells. Our results reveal important heterogeneity across the surface of the clusters. Maps at high resolution reveal variable mechanical properties at cell–cell junctions. Across the different maps obtained (eight in total), cells near the border of the cluster appear slightly stiffer than those at the cluster centre. These preliminary results may suggest that cell phenotype depends on the localisation within the cluster, although additional experiments should be carried out to confirm this claim.

Most studies of membrane tether formation have so far applied pulling velocities ($\overset{`}{L}$ < 100 μm/s), in which the force required to extrude the tether is dominated by the friction of the lipids with the membrane proteins anchored to the cytoskeleton. This limited range of pulling velocities was likely due to the slow response time of common piezoelectric elements [5] and, if AFM‐based, because of cantilevers with high viscous drag coefficient [5, 53, 54]. The possibility of using ultrashort cantilevers with low viscous drag on our HS‐AFM platform allowed us to reach pulling velocities up to ~6 mm/s regime, well above the predicted upper limit of the friction dominated regime.

As described by Brochard–Wyart [51], in the friction dominated regime, the force scales roughly with the cubic root of the pulling velocity as follows:

(1) $$f^{3}+ff_{0}^{2}={\left(2\pi \right)}^{3}2\kappa^{2}\nu \eta \overset{`}{L}\ln\left(R/r_{t}\right)$$

where $f_{0}$ is the static tether force, κ is the bending modulus of the membrane, ν is the surface density of membrane proteins bound to the cytoskeleton, η is the surface viscosity (or $1/\xi^{2}$, being ξ the average distance between proteins) and R and $r_{t}$ are the cell and membrane tether radii, respectively. Our measured tether forces increased with the pulling velocity and were relatively well described by Equation (1), suggesting that the process was still dominated by friction, even at the highest velocities. This may be explained by the shear geometry in the application of force to the bonds, which may reduce the actual applied force, effectively increasing the upper limit of $\overset{`}{L}$. Future developments of our control algorithms will allow pulling tethers with a more physiologically relevant geometry, by moving laterally while retracting in the *z*‐direction, although this has shown not to modify tether formation forces in neutrophils [55]. Equation (1) fitted the results reasonably well, considering the four orders of magnitude of dynamic range, and predicted a static force of ∼44 pN. Our previous work on the same cell line reported tether forces around 30 pN after latrunculin A treatment, a lower limit for the static force [5]. This difference may suggest other mechanisms behind the dynamic tether formation in monocytic cells.

In her work, Brochard–Wyart suggested that, at high velocities ($\overset{`}{L}$ > 100 μm/s), the friction force is expected to overcome the force required to break the bonds formed by membrane proteins and the actin cytoskeleton, leading bond rupture, generally described by a Bell‐Evans, activated regime in which the force grows with the logarithm of the loading rate [56]. Interestingly, although not observed before in other tether forming systems, this model seems to fit better our experimental results. If this were the mechanism, tether forces would be dominated by the shear breaking of the membrane‐cytoskeleton bonds over the whole dynamic range, much lower than the Brochard–Wyart predicted limit. This may be explained by a high membrane viscosity in monocytes, which would decrease the upper limit of the friction dominated regime. The localisation of VLA‐4 integrins at the tip of protruding microvilli may induce a tether extraction geometry in which lipids flow parallel to the pulling velocity, as opposed to perpendicular. This behaviour may be more prone to energy dissipation, helping monocytes transition from capture to rolling during the leucocyte adhesion cascade. Besides that, disruption of the bonds between membrane proteins and the cytoskeleton may facilitate the formation of slings, membrane tethers including adhesion molecules along their length, reported previously on neutrophils [3, 57]. While our results are specific to the VLA‐4/VCAM‐1 mediated tether formation, our approach opens the door to understanding other leucocyte adhesion molecules and their behaviour at physiologically relevant velocities.

## 5 Conclusion

In this work, we have developed a HS‐AFM platform with an extended *z*‐range, enabling rapid acquisition of long‐range force curves at millimetre‐per‐second velocities. The integration of a custom GUI and FPGA‐based control system facilitated synchronised actuation and data acquisition, while the implementation of fast ‘moustache’ algorithms and predictive strategy significantly reduced acquisition time and data density of force measurements. Using this system, we successfully acquired force maps over 40‐μm thick cell clusters in approximately 9–10 min, revealing the capability for rapid, quantitative mechanical mapping of heterogeneous and thick biological samples. The analysis of these datasets using PyFMlab allowed us to extract topography, scaling elastic modulus and fluidity index, showcasing the system's effectiveness in characterising complex biological structures. While the biological relevance of the maps is limited, it provides a proof of concept of the developed algorithms applicable to more biologically relevant experiments. Furthermore, we explored cell adhesion in monocytic cells by measuring the forces required to extract membrane tethers with functionalised ultrasmall cantilevers, reaching a wide range of retract velocities from 3 to 6000 μm/s. Our results provided insights into tether formation behaviour at physiologically relevant velocities, highlighting the potential of our platform for studying dynamic cellular processes.

The widespread adoption of FPGA technology in AFM control software enables the development of advanced algorithms similar to those presented here. Potential enhancements include real‐time prediction of force setpoints to mitigate overshooting of piezo movement, real‐time detection of successful unbinding events, and dynamic adjustment of the scanning area in response to changes in the sample properties. We anticipate that this trend will accelerate as proprietary control software is replaced by open‐source alternatives.

## Author Contributions

Q.Z. and F.R. supervised research. L.V., Y.S., M.E. and A.C. conducted HS‐AFM measurements. L.V., Q.Z. and F.R. developed the control algorithms. L.V. and Y.S. analysed the data with F.R. L.V. and J.R.‐R. developed and calibrated the long range *z*‐stage. L.V., Q.Z. and F.R. wrote the paper with the contributions of all the authors.

## Funding

This project received funding from the European Research Council (ERC) under the European Union's Horizon 2020 (772257 and 101189381 to F.R.), from the French Agence Nationale de la Recherche (ANR‐23‐CE30‐0048 BioHETER to F.R.), from the National Science Foundation (NSF‐PHY‐2412551 and NSF‐PFI‐2234449 to Q.Z.) and from Rutgers TechAdvance (to Q.Z.). We also acknowledge Inserm and Aix‐Marseille University for regular support. Electron microscopy experiments were carried out at the PICsL‐FBI core electron microscopy facility (Nicolas Brouilly, IBDM, AMU‐Marseille UMR 7288), a member of the national infrastructure France‐BioImaging supported by the Agence Nationale de la Recherche (ANR‐10‐INBS‐0004).

## Ethics Statement

Ethics approval statement is not applicable.

## Consent

Consent statement is not applicable or needed for this study.

## Conflicts of Interest

The authors declare no conflicts of interest.

## Data Availability

The data that support the findings of this study are available from the corresponding author upon reasonable request.

## Endnotes

^1^
The name moustache comes from the curved shape of the fast approach.
